# Research on the Rapid and Accurate Positioning and Orientation Approach for Land Missile-Launching Vehicle

**DOI:** 10.3390/s151026606

**Published:** 2015-10-20

**Authors:** Kui Li, Lei Wang, Yanhong Lv, Pengyu Gao, Tianxiao Song

**Affiliations:** 1 School of Electronic and Information Engineering, Beihang University, Beijing 100191, China; E-Mail: eric.lee_buaa@buaa.edu.cn; 2 School of Instrument Science and Opto-electronics Engineering, Beihang University, Beijing 100191, China; E-Mails: yanhonglv@buaa.edu.cn (Y.L.); gaodiao008@163.com (P.G.); songtianxiao_buaa@126.com (T.S.)

**Keywords:** land vehicle, positioning and orientation, biaxial optical detection platform

## Abstract

Getting a land vehicle’s accurate position, azimuth and attitude rapidly is significant for vehicle based weapons’ combat effectiveness. In this paper, a new approach to acquire vehicle’s accurate position and orientation is proposed. It uses biaxial optical detection platform (BODP) to aim at and lock in no less than three pre-set cooperative targets, whose accurate positions are measured beforehand. Then, it calculates the vehicle’s accurate position, azimuth and attitudes by the rough position and orientation provided by vehicle based navigation systems and no less than three couples of azimuth and pitch angles measured by BODP. The proposed approach does not depend on Global Navigation Satellite System (GNSS), thus it is autonomous and difficult to interfere. Meanwhile, it only needs a rough position and orientation as algorithm’s iterative initial value, consequently, it does not have high performance requirement for Inertial Navigation System (INS), odometer and other vehicle based navigation systems, even in high precise applications. This paper described the system’s working procedure, presented theoretical deviation of the algorithm, and then verified its effectiveness through simulation and vehicle experiments. The simulation and experimental results indicate that the proposed approach can achieve positioning and orientation accuracy of 0.2 m and 20″ respectively in less than 3 min.

## 1. Introduction

Modern war is having increasingly higher requirements for weapon launching systems. To improve the weapon’s survival capability in the battlefield, it needs weapon launching systems not only to be capable of accurate aiming at the objects, but also to be able to quickly respond and to have high flexibility, agility, and reliability [1]. This means that a weapon launching system must be capable of acquiring its accurate position, attitudes and orientation rapidly [2]. Consequently, designing an approach which can obtain the accurate launching reference information rapidly for launch of weapons is of great significance and value for improvement of ground artillery’s survival capability, rapid response capability and maneuvering capability.

To achieve this goal, lots of work has been done in the past. Theodolite, Inertial Navigation System (INS), odometer, land markers correct, zero-velocity correct, Global Navigation Satellite System (GNSS) are the most commonly used methods and equipment [3,4,5,6]. Some multiple integrated approaches such as INS/GNSS integration, INS and odometer integration (INS/DR) are also studied and applied [7,8,9].

Theodolite can provide high performance orientation information by itself [3], but its operation is usually laborious and needs relatively long time, thus it is not beneficial for battlefield survival. INS is autonomous, which does not need external measuring information and has strong anti-interference ability [10]. However, INS’s positioning and orientation errors increase with time [11]. If INS has been working for a relatively long time, its reference information is hard to satisfy accurate launching requirements. GNSS can achieve high positioning results, and its navigation errors do not divergent with time, but during wartime, it has the risk of being interfered and cannot provide high precision orientation and attitude reference [12]. Integration of INS and GNSS can combine both systems’ advantages [13,14]. However, it would lose some autonomy since GNSS is involved in real-time. Odometer is used to measure speed and distance of the vehicle moving on the ground. It cannot be used independently for positioning but can be used with INS for Dead Reckoning (DR) [15]. INS/DR integration approach is widely used in land weapon launching applications since it can provide relatively high precision reference information as well as keep autonomy [16]. However, for most approaches mentioned above, if precise orientation and attitudes are needed, high performance INS is necessary, which is always expensive and needs much preparation time.

Based on this background, the paper proposed a new solution. The approach only needs land vehicle based INS or INS/DR to provide rough position and orientation as iterative initial value, thus it does not have high performance requirements even in high precision applications. The new solution uses a vehicle based BODP to aim at and lock no less than three pre-set cooperative targets. Since the cooperative targets are set beforehand, their accurate coordinates can be measured by differential GNSS (DGNSS). After acquiring measurements from BODP, the vehicle’s accurate position and orientation can be calculated. The proposed approach does not depend on GNSS and is autonomous. This paper described the basic principle of the system. Section 2 describes the system working procedure, and presents theoretical deviation of the algorithm. Simulations results under different conditions are given in Section 3. Vehicle experiments are carried out and experiment results are presented and analyzed in Section 4, and conclusions are drawn in Section 5.

## 2. Principle of the Proposed Approach

### 2.1. System Working Procedure

The vehicle positioning and orientation system contains several subsystems, including INS/DR integration system, a vehicle based BODP, and several cooperative targets arranged around the launching areas. The vehicle starts from home and stops at any point and to any direction in the selected launching areas. The system uses the targets’ coordinates and vehicle’s coordinates provided by INS/DR to calculate targets’ rough azimuth and pitch angle relative to vehicle. Then, it rotates BODP to search and lock the targets, and output the targets’ precise azimuth and pitch angles relative to vehicle. After acquiring no less than three couples of azimuth and pitch angles, the system could calculate vehicle’s accurate horizontal position and orientation, and then, the vehicle’s accurate height and attitudes. The following section would give detailed positioning, orientation and attitudes determination algorithms that proposed.

### 2.2. Orientation and Horizontal Position Determination Algorithm

**Figure 1:**
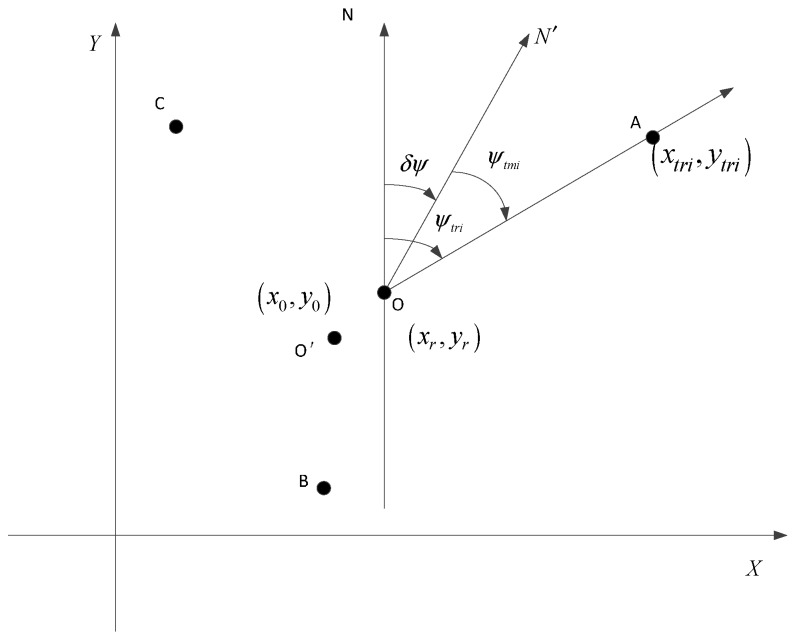
Orientation and horizontal position determination diagram.

Establish a rectangular coordinate system shown in Figure 1A–C are cooperative targets, and O is the vehicle’s parking position. Use $\left(x_{tmi},y_{tmi},z_{tmi}\right)$, i = 1, 2, 3 to denote the targets’ coordinates which are measured by DGNSS, and $\left(x_{tri},y_{tri},z_{tri}\right)$ i = 1, 2, 3 to denote the targets’ true coordinates. The vehicle based INS/DR provides vehicle’s coordinates as $\left(x_{0},y_{0},z_{0}\right)$, while the vehicle’s real coordinates are $\left(x_{r},y_{r},z_{r}\right)$. The direction ON denotes true north of geographic coordinate system, and $ON^{\prime }$ denotes north direction given by INS/DR. Use δψ to denote the difference between ON and $ON^{\prime }$. The true attitude and azimuth of the vehicle are ${\text{θ}}_{\text{r}}{\text{,γ}}_{\text{r}}{\text{,ψ}}_{\text{r}}$ respectively, and θ,γ,ψ are vehicle’s attitude and azimuth provided by INS/DR. The BODP aiming at the targets A, B, C and output three couples of azimuth and pitch angles denoted by $\left(\psi_{tm1},\phi_{m1}\right)$, $\left(\psi_{tm2},\phi_{m2}\right)$ and $\left(\psi_{tm3},\phi_{m3}\right)$, while the true angles are denoted by $\left(\psi_{tr1},\phi_{r1}\right)$, $\left(\psi_{tr2},\phi_{r2}\right)$ and $\left(\psi_{tr3},\phi_{r3}\right)$.

From geometric relations shown in Figure 1, we can get the following equation:

(1) $$\left(x_{tri}-x_{r}\right)\cos{\text{ψ}}_{tri}=\left(y_{tri}-y_{r}\right)\sin{\text{ψ}}_{tri},\;\text{i}=1,\;2,\;3$$

Linearize Equation (1) by Taylor expansion at $\left(x_{0},y_{0},x_{tmi},y_{tmi}\right)$ as follows:

(2) $$\begin{array}{lll} {\text{ψ}}_{tri} & = & {\text{ψ}}_{tmi}+\text{δψ}\\ & = & {\text{ψ}}_{tri}{|}_{\left(x_{0},y_{0},x_{tmi},y_{tmi}\right)}+\frac{\partial {\text{ψ}}_{tri}}{\partial x_{r}}{|}_{\left(x_{0},y_{0},x_{tmi},y_{tmi}\right)}\text{δ}x_{0}+\frac{\partial {\text{ψ}}_{tri}}{\partial y_{r}}{|}_{\left(x_{0},y_{0},x_{tmi},y_{tmi}\right)}\text{δ}y_{0},\;\text{i}=1,\;2,\;3\\ & & +\frac{\partial {\text{ψ}}_{tri}}{\partial x_{tri}}{|}_{\left(x_{0},y_{0},x_{tmi},y_{tmi}\right)}\text{δ}x_{tmi}+\frac{\partial {\text{ψ}}_{tri}}{\partial y_{tri}}{|}_{\left(x_{0},y_{0},x_{tmi},y_{tmi}\right)}\text{δ}y_{tmi} \end{array}$$

where ${\text{ψ}}_{tri}{|}_{\left(x_{0},y_{0},x_{tmi},y_{tmi}\right)}$ is the value of ${\text{ψ}}_{tri}$ at $\left(x_{0},y_{0},x_{tmi},y_{tmi}\right)$, i = 1, 2, 3, and

$$\frac{\partial {\text{ψ}}_{tri}}{\partial x_{r}}=\frac{-1}{\left(y_{tri}-y_{r}\right)+\left(x_{tri}-x_{r}\right)\tan{\text{ψ}}_{tri}}\;\frac{\partial {\text{ψ}}_{tri}}{\partial y_{r}}=\frac{1}{\left(x_{tri}-x_{r}\right)+\left(y_{tri}-y_{r}\right)\cot{\text{ψ}}_{tri}}$$

$$\frac{\partial {\text{ψ}}_{tri}}{\partial x_{tri}}=\frac{1}{\left(y_{tri}-y_{r}\right)+\left(x_{tri}-x_{r}\right)\tan{\text{ψ}}_{tri}}\;\frac{\partial {\text{ψ}}_{tri}}{\partial y_{tri}}=\frac{-1}{\left(x_{tri}-x_{r}\right)+\left(y_{tri}-y_{r}\right)\cot{\text{ψ}}_{tri}}$$

Since coordinates of the cooperative targets A, B and C are acquired by precise measurement, compared to $\text{δ}x_{0}$ and $\text{δ}y_{0}$, $\text{δ}x_{tmi}$ and $\text{δ}y_{tmi}$ can be ignored. Then, Equation (2) can be simplified as follows:

(3) $${\text{ψ}}_{tmi}+\text{δψ}\dot{=}{\text{ψ}}_{tri}{|}_{\left(x_{0},y_{0},x_{tmi},y_{tmi}\right)}+\frac{\partial {\text{ψ}}_{tri}}{\partial x_{r}}{|}_{\left(x_{0},y_{0},x_{tmi},y_{tmi}\right)}\text{δ}x_{0}+\frac{\partial {\text{ψ}}_{tri}}{\partial y_{r}}{|}_{\left(x_{0},y_{0},x_{tmi},y_{tmi}\right)}\text{δ}y_{0},\;\text{i}=1,\;2,\;3$$

If three cooperative targets are measured, the corresponding equations can be written as following:

(4) $$\Delta L_{H}=H_{H}\Delta E_{H},\;\text{i}=1,\;2,\;3$$

where

$$\Delta L_{H}=\left(\begin{array}{l} \psi_{tm1}-\psi_{tr1}{|}_{\left(x_{0},y_{0},x_{tm1},y_{tm1}\right)}\\ \psi_{tm2}-\psi_{tr2}{|}_{\left(x_{0},y_{0},x_{tm2},y_{tm2}\right)}\\ \psi_{tm3}-\psi_{tr3}{|}_{\left(x_{0},y_{0},x_{tm3},y_{tm3}\right)} \end{array}\right),\;\Delta E_{H}=\left(\begin{array}{l} \text{δ}x_{0}\\ \text{δ}y_{0}\\ \text{δψ} \end{array}\right)\text{ and }A_{H}=\left(\begin{array}{ccc} \frac{\partial \psi_{tr1}}{\partial x_{r}}{|}_{\left(x_{0},y_{0},x_{tm1},y_{tm1}\right)} & \frac{\partial \psi_{tr1}}{\partial y_{r}}{|}_{\left(x_{0},y_{0},x_{tm1},y_{tm1}\right)} & -1\\ \frac{\partial \psi_{tr2}}{\partial x_{r}}{|}_{\left(x_{0},y_{0},x_{tm2},y_{tm2}\right)} & \frac{\partial \psi_{tr2}}{\partial y_{r}}{|}_{\left(x_{0},y_{0},x_{tm2},y_{tm2}\right)} & -1\\ \frac{\partial \psi_{tr3}}{\partial x_{r}}{|}_{\left(x_{0},y_{0},x_{tm3},y_{tm3}\right)} & \frac{\partial \psi_{tr3}}{\partial y_{r}}{|}_{\left(x_{0},y_{0},x_{tm3},y_{tm3}\right)} & -1 \end{array}\right)$$

Then, the initial horizontal position and orientation errors can be calculated by the following Equation:

(5) $$\Delta E_{H}={\left({A_{H}}^{T}A_{H}\right)}^{-1}{A_{H}}^{T}\Delta L_{H},\;\text{i}=1,\;2,\;3$$

After calculating $\Delta E_{H}$, use it to update and correct INS/DR positioning result (x_0_,y_0_) and orientation error δψ, recalculate each target’s true azimuth ${\text{ψ}}_{tmi}$ relative to vehicle, and then use the corrected positioning and orientation results to do the same procedure, until each iteration correction $\Delta E_{H}$ is within the expected accuracy. By finishing the iteration, the horizontal position and orientation of the vehicle is accurately determined.

### 2.3. Attitudes and Height Determination Algorithm

In the proposed approach, the vehicle’s accurate attitudes and height determination algorithm is on the basis of orientation and horizontal position determination results. After acquiring the accurate horizontal position and orientation results in the previous section, their errors are assumed to be negligible when determining the vehicle’s attitudes and height.

**Figure 2:**
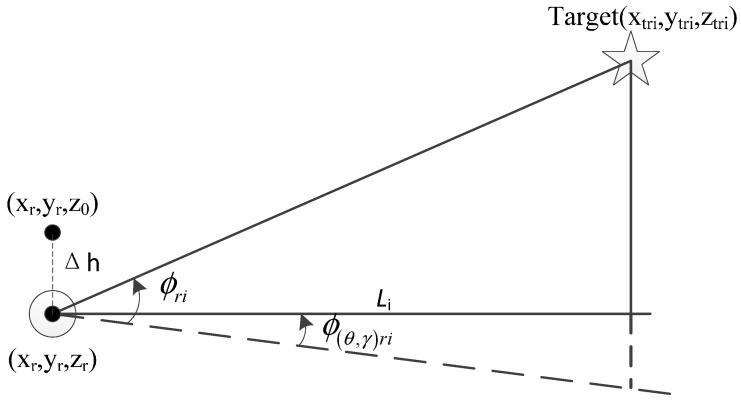
Relationship of vehicle and target’s position and measurements.

The relationship of vehicle and targets’ position and angles measurements are shown in Figure 2:

Where $L_{i}$ is the horizontal distance between vehicle and the *i*th target. $\phi_{(\theta ,\gamma )ri}$ is the true angle from optical axis of BODP when rotating around the vertical axis to the direction just below the target to the horizontal plane.

It can be seen from Figure 2 that:

(6) $$\tan(\phi_{ri}-\phi_{(\theta ,\gamma )ri})=\frac{z_{ti}-z_{r}}{L_{i}},\;\text{i}=1,\;2,\;3$$

From the Euler angles rotation relationship and Figure 2, the following equation can be derived:

(7) $$\sin\phi_{(\theta ,\gamma )ri}=\sin{\text{γ}}_{r}\sin\text{ψ}+\cos{\text{γ}}_{r}\sin{\text{θ}}_{r}\cos\text{ψ},\;\text{i}=1,\;2,\;3$$

In land vehicle missile-launching applications, usually relatively firm and flat areas are selected as launching point, and the attitudes of the vehicle are usually small angles. If attitudes errors provided by INS/DR are Δθ and Δγ, that is, ${\text{θ}}_{r}=\text{θ}+\Delta \text{θ},{\text{γ}}_{r}=\text{γ}+\Delta \text{γ}$, then Equation (7) can be simplified as:

(8) $$\begin{array}{ll} \phi_{(\text{θ,γ})ri} & =\sin(\text{γ}+\Delta \text{γ})\sin\text{ψ}+\cos(\text{γ}+\Delta \text{γ})\sin(\text{θ}+\Delta \text{θ})\cos\text{ψ}\\ & \dot{=}\text{θ}\cos{\text{ψ}}_{i}+\text{γ}\sin{\text{ψ}}_{i}+\Delta \text{θ}\cos{\text{ψ}}_{i}+\Delta \text{γ}\sin{\text{ψ}}_{i} \end{array},\;\text{i}=1,\;2,\;3$$

The BODP measured $\phi_{(\theta ,\gamma )ri}$ is denoted by $\phi_{(\theta ,\gamma )mi}$, which is given by:

(9) $$\phi_{(\text{θ,γ})mi}=\text{θ}\cos{\text{ψ}}_{i}+\text{γ}\sin{\text{ψ}}_{i},\;\text{i}=1,\;2,\;3$$

Use $\Delta \phi_{(\theta ,\gamma )i}$ to denote error of $\phi_{(\theta ,\gamma )ri}$, and $\Delta \phi_{(\theta ,\gamma )i}$ is given by:

(10) $$\Delta \phi_{(\theta ,\gamma )i}=\Delta \text{θ}\cos{\text{ψ}}_{i}+\Delta \text{γ}\sin{\text{ψ}}_{i},\;\text{i}=1,\;2,\;3$$

From Equations (8)–(10), it can be seen that:

(11) $$\phi_{(\theta ,\gamma )ri}=\phi_{(\theta ,\gamma )mi}+\Delta \phi_{(\theta ,\gamma )i},\;\text{i}=1,\;2,\;3$$

Assume $\phi_{i}=\phi_{ri}-\phi_{(\theta ,\gamma )mi}$, where the pitch angle $\phi_{ri}$ is measured by BODP, then the following equation can be derived:

(12) $$\tan(\phi_{ri}-\phi_{(\theta ,\gamma )ri})=\tan(\phi_{i}-\Delta \phi_{(\theta ,\gamma )i})=\frac{\tan\phi_{i}-\tan\Delta \phi_{(\theta ,\gamma )i}}{1+\tan\phi_{i}\tan\Delta \phi_{(\theta ,\gamma )i}}=\frac{z_{ti}-z_{0}}{L_{i}}+\frac{\Delta h}{L_{i}},\;\text{i}=1,\;2,\;3$$

Since $\Delta \phi_{(\theta ,\gamma )i}$ is small angle, $\tan\Delta \phi_{(\theta ,\gamma )i}≐\Delta \phi_{(\theta ,\gamma )i}=\Delta \text{θ}\cos{\text{ψ}}_{i}+\Delta \text{γ}\sin{\text{ψ}}_{i}$, thus the following equation can be derived:

(13) $$\tan\phi_{i}-\frac{z_{ti}-z_{0}}{L_{i}}=\frac{\Delta h}{L_{i}}+\Delta \theta \left(1+\frac{z_{ti}-z_{0}}{L_{i}}\tan\phi_{i}\right)\cos\psi_{i}+\Delta \gamma \left(1+\frac{z_{ti}-z_{0}}{L_{i}}\tan\phi_{i}\right)\sin\psi_{i},\;\text{i}=1,\;2,\;3$$

If three cooperative targets are measured, the corresponding equations can be written as following:

(14) $$\Delta L_{V}=A_{V}\Delta E_{V},\;\text{i}=1,\;2,\;3$$

where,

$$\Delta L_{V}={\left[\tan\phi_{1}-\frac{z_{t1}-z_{0}}{L_{1}}\tan\phi_{2}-\frac{z_{t2}-z_{0}}{L_{2}}\tan\phi_{3}-\frac{z_{t3}-z_{0}}{L_{3}}\right]}^{T}$$

$$A_{V}=\left[\begin{array}{l} \left(1+\frac{z_{t1}-z_{0}}{L_{1}}\tan\phi_{1}\right)\cos\psi_{1}\left(1+\frac{z_{t1}-z_{0}}{L_{1}}\tan\phi_{1}\right)\sin\psi_{1}\frac{1}{L_{1}}\\ \left(1+\frac{z_{t2}-z_{0}}{L_{2}}\tan\phi_{2}\right)\cos\psi_{2}\left(1+\frac{z_{t2}-z_{0}}{L_{2}}\tan\phi_{2}\right)\sin\psi_{2}\frac{1}{L_{2}}\\ \left(1+\frac{z_{t3}-z_{0}}{L_{3}}\tan\phi_{3}\right)\cos\psi_{3}\left(1+\frac{z_{t3}-z_{0}}{L_{3}}\tan\phi_{3}\right)\sin\psi_{3}\frac{1}{L_{3}} \end{array}\right]$$

$$\Delta E_{V}={\left[\Delta \theta \Delta \gamma \Delta h\right]}^{T}$$

Then, the initial height and attitudes errors can be calculated by the following equation:

(15) $$\Delta E_{V}={\left({A_{V}}^{T}A_{V}\right)}^{-1}{A_{V}}^{T}\Delta L_{V},\;\text{i}=1,\;2,\;3$$

After calculating $\Delta E_{V}$, use it to update and correct INS/DR height and attitudes errors. And then use the corrected results to do the same procedure, until each iteration correction $\Delta E_{V}$ is within the expected accuracy. By finishing the iteration, the vehicle’s height and attitudes can be accurately determined.

## 3. Simulation Results

### 3.1. Orientation and Horizontal Position Determination Simulation

**Figure 3:**
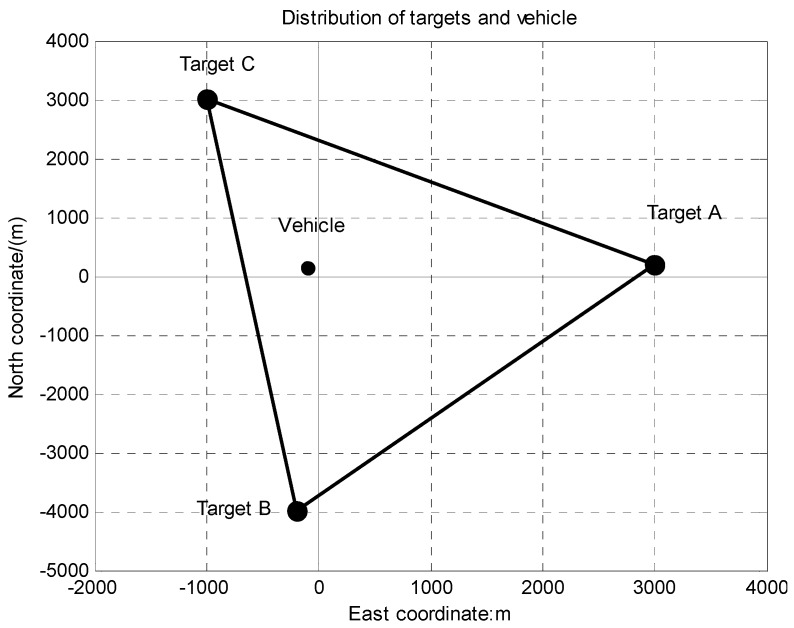
Distribution of targets and vehicle.

Consider the orientation and horizontal position determination algorithm simulation first. Establish the measurement rectangular coordinate system as shown in Figure 3. Assume that the true horizontal coordinates of three targets and the vehicle are (3000 m, 200 m), (−200 m, −4000 m), (−1000 m, 3000 m) and (−100 m, 150 m) respectively.

**Figure 4:**
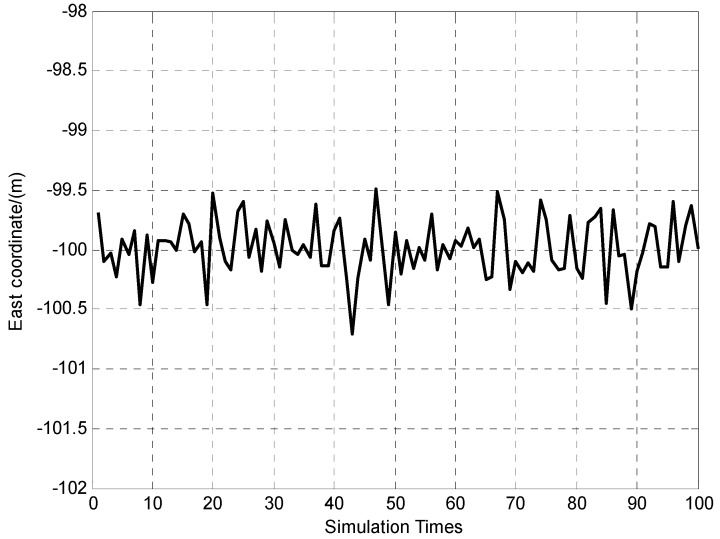
100 times of east coordinate determination results.

**Figure 5:**
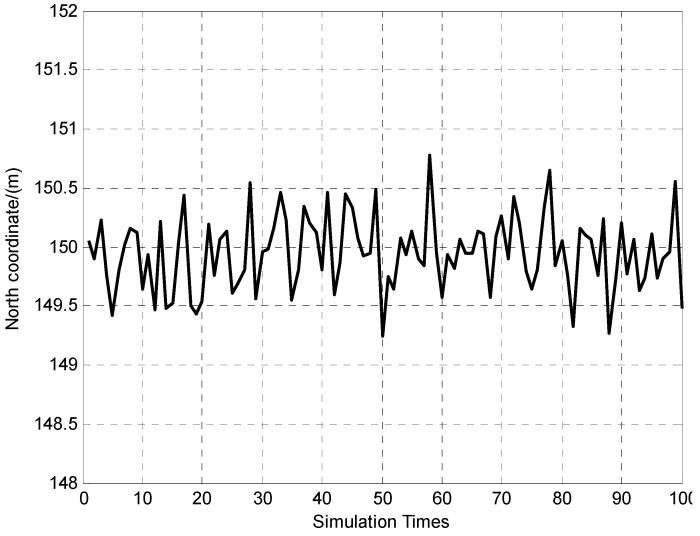
100 times of north coordinate determination results.

**Figure 6:**
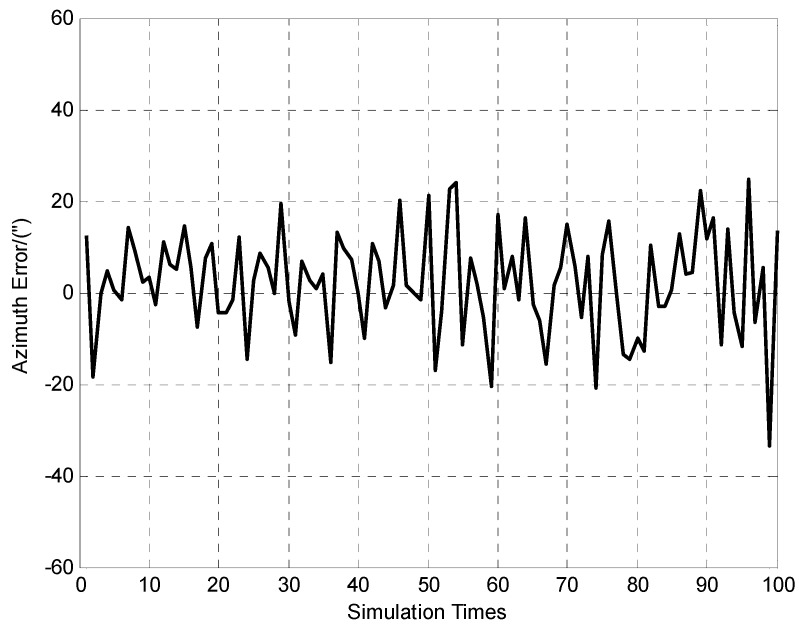
100 times of azimuth error determination results.

Since cooperative targets are set beforehand, their coordinates can be carefully calibrated. In the following simulation, the cooperative targets’ coordinates measuring errors are assumed to be 0.3 m (1σ). Positioning error and azimuth error of vehicle based INS/DR are assumed to be 300 m (1σ) and 0.5° (1σ). BODP azimuth and pitch angles measuring errors are assumed to be 5″ (1σ) when aiming at and locking the targets. Apply the proposed orientation and horizontal position determination algorithm under the above conditions and simulate 100 times. The simulation results are shown in Figure 4, Figure 5 and Figure 6.

It can be seen from Figure 4 and Figure 5 that the positioning errors of the vehicle are within ±1 m (Standard deviation of east and north coordinate errors are 0.2 m and 0.3 m respectively). The Figure 6 indicates that azimuth error of the vehicle is within ±40″, and the standard deviation is 11.1″.

### 3.2. Attitudes and Height Determination Simulation

**Figure 7:**
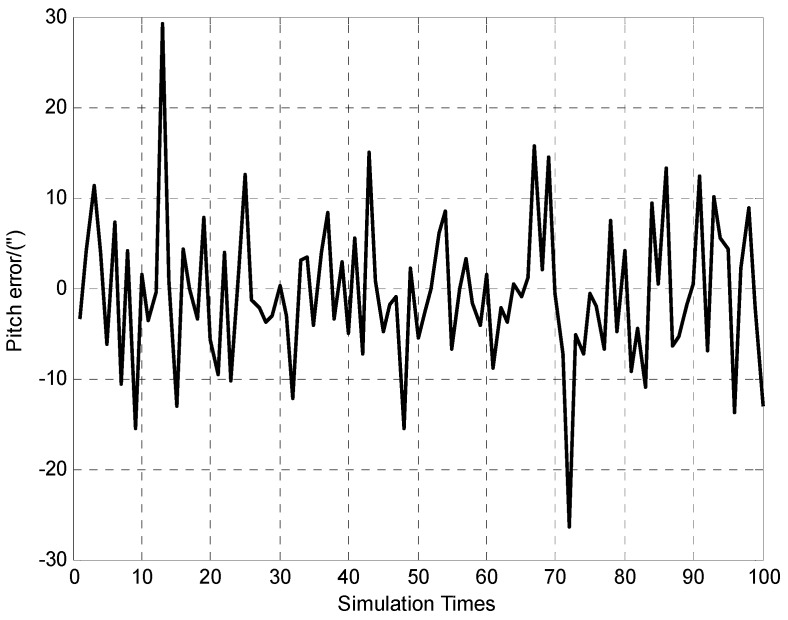
100 times of pitch error determination results.

**Figure 8:**
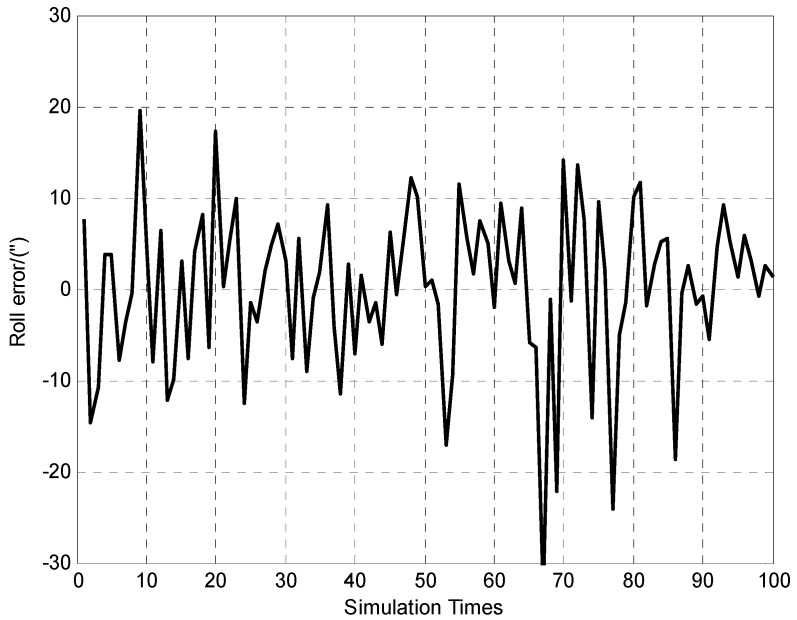
100 times of roll error determination results.

**Figure 9:**
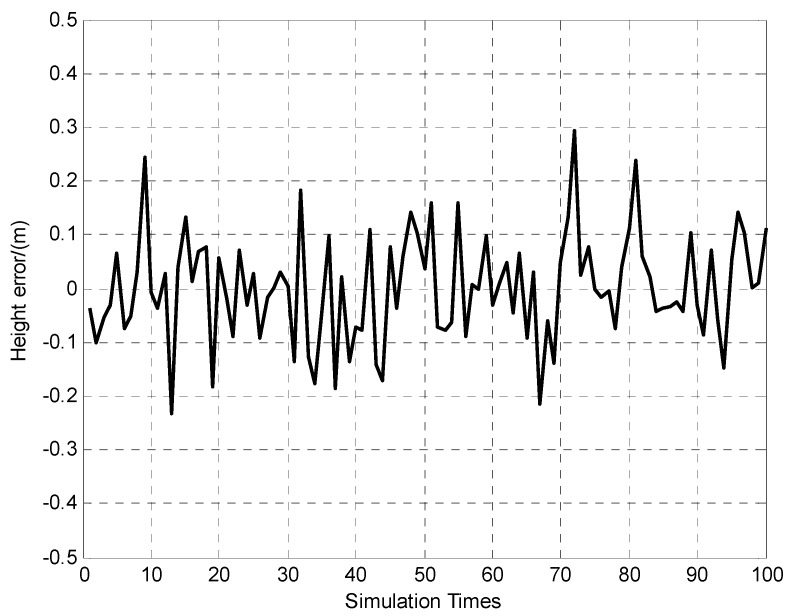
100 times of height error determination results.

Assume that the true height of the three cooperative targets and vehicle are 500 m, 480 m, 800 m and 60 m respectively. The height measuring errors of the targets are assumed to be 0.3 m (1σ). The vehicle’s true pitch and roll attitudes are 4° and 3°, while, the attitudes error and height error of the vehicle based INS/DR are 0.2° (1σ) and 200 m respectively. In the theoretical deviation for attitudes and height determination algorithm, the vehicle’s orientation and horizontal position errors were assumed to be zero. However, the horizontal position and orientation errors are inevitable. Consequently, in vehicle’s attitudes and height determination simulations here, we add horizontal position errors of 0.3 m (1σ) and orientation error of 20″ (1σ), which is the same accuracy as previous simulation results. Apply the proposed attitudes and height determination algorithm under the above conditions and simulate 100 times. The simulation results are shown in Figure 7, Figure 8 and Figure 9.

It can be seen from Figure 7 and Figure 8 that the vehicle’s attitudes determination errors are within ±30″ (Standard deviation of pitch and roll errors are 7.9″ and 8.8″ respectively). The Figure 9 indicates that vehicle’s height determination error is within ±0.3 m, and the standard deviation is 0.1 m.

## 4. Vehicle Experiments and Analysis

**Figure 10:**
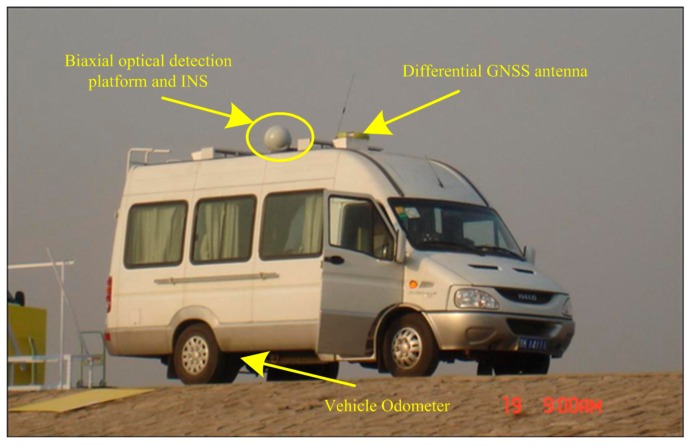
Experimental vehicle and relative equipment.

The proposed positioning and orientation system mainly consist of vehicle based equipment and cooperative targets. The vehicle-based equipment includes INS/DR integration system, a DGNSS receiver and a BODP. The experimental vehicle and relative equipment are shown in Figure 10.

In the experiment, the odometer is chosen with 1024 pulse output when vehicle’s wheel rounding a whole circle. And the positioning accuracy of INS/DR is about 0.001 times of distance that vehicle moves. The BODP pitch and azimuth angles measuring accuracy are about 5″ when aiming at and locking the cooperative targets. The DGNSS positioning accuracy is about 0.02 m (1σ).

**Figure 11:**
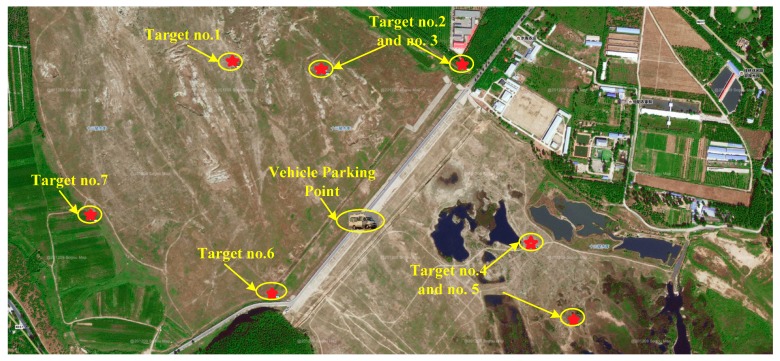
Distribution of the cooperative targets and vehicle.

The experimental area is selected in the suburb of Beijing city. Seven cooperative targets are settled in the area, which are numbered as target no.1, 2…and 7. The distribution of the cooperative targets and vehicle parking point are shown in Figure 11. The precise coordinates of all cooperative targets are measured by DGNSS. In addition, the vehicle is also equipped with DGNSS to provide reference position for algorithm evaluation.

The experimental vehicle is driven to the parking point from the starting point several kilometers away. Then, use the vehicle based DGNSS receiver to measure the vehicle’s precise position as reference. The DGNSS position measuring results of the targets and the vehicle parking point are given in Table 1.

**Table 1:** Positions of the targets and parking points.

	Longitude (°)	Latitude (°)	Height (m)
Parking point	116.2469562	40.2683542	70.90
Target no.1	116.2428290	40.2722016	79.20
Target no.2	116.2454469	40.2717759	79.17
Target no.3	116.2496097	40.2713175	84.10
Target no.4	116.2515194	40.2676992	83.89
Target no.5	116.2546308	40.2661110	80.13
Target no.6	116.2443096	40.2664876	102.16
Target no.7	116.2395675	40.2683541	89.23

After the BODP aims at and lock the targets, it would output azimuth and pitch angles relative to vehicle. Apply the proposed positioning and orientation approach to the equipment measurements, and then give the calculation results. In the experiment, the targets no. 2, 5, 7 were selected and the same experiment procedure was done seven times. Every time a new set of measuring parameters were acquired. Then, apply the proposed approach to seven sets of measurements and the results are given in Table 2.

**Table 2:** Positioning and orientation results

	Longitude (°)	Latitude (°)	Azimuth (°)	Height (m)	Pitch (°)	Roll (°)
1	116.2469543	40.2683567	39.0451	71.10	−0.0677	−2.0003
2	116.2469520	40.2683559	39.0475	71.08	−0.0628	−1.9984
3	116.2469550	40.2683559	39.0404	71.19	−0.0677	−2.0002
4	116.2469540	40.2683561	39.0441	71.20	−0.0628	−1.9978
5	116.2469541	40.2683569	39.0315	71.06	−0.0661	−2.0002
6	116.2469534	40.2683565	39.0450	71.92	−0.0669	−2.0006
7	116.2469518	40.2683555	39.0412	71.03	−0.0632	−1.9986
Mean	116.2469535	40.2683562	39.0421	71.23	−0.0653	−1.9994
Std	0.11 m	0.06 m	19.0″	0.31 m	8.3″	4.1″

It can be seen from Table 2 that in the seven experiments, the standard deviation of horizontal positioning and height errors are about 0.1 m and 0.3 m, and attitudes (pitch and roll) and orientation errors are about 8″ and 20″ respectively. The statistical results indicate that the proposed approach displays good repeatability. To further verify the effectiveness of the proposed approach, five different sets of targets were selected to form different groups of measurements. The five groups of targets combinations are targets no. 2,5,7 combination, targets no. 1,4,6 combination, targets no. 3,4,7 combination, targets no. 1,3,5 combination, and targets no. 2,4,6 combination respectively. The proposed approach was applied to the five combinations of targets, and the calculation results are given in Table 3.

**Table 3:** Positioning and orientation results using different combination of targets

Targets no.	Longitude (°)	Latitude (°)	Azimuth (°)	Height (m)	Pitch (°)	Roll (°)
No. 2,5,7	116.2469534	40.2683571	39.0409	71.11	−0.0661	−2.0000
No. 1,4,6	116.2469549	40.2683563	39.0514	70.95	−0.0715	−2.0035
No. 3,4,7	116.2469539	40.2683560	39.0331	70.93	−0.0709	−2.0021
No. 1,3,5	116.2469560	40.2683555	39.0521	71.11	−0.0642	−1.9982
No. 2,4,6	116.2469546	40.2683567	39.0310	70.70	−0.0751	−2.0063
Mean	116.2469546	40.2683563	39.0417	70.96	−0.0696	−2.0020
Reference	116.2469562	40.2683542		70.90		
Max offset	0.24 m	0.32 m	38.5″	0.21 m	19.8″	15.5″

In Table 3, the vehicle’s position provided by DGNSS is listed as reference. It can be seen that compared to the reference, the approach can achieve positioning accuracy of about 0.3 m maximum offset by using different sets of targets. Since there isn’t any other high performance azimuth and attitude measuring equipment in the vehicle during the experiments, the true azimuth and attitude reference are not acquired. In this circumstance, the mean attitude determination results are used as reference and only repeatability when using different groups of targets are evaluated. In addition, the results show that the azimuth maximum offset relative to mean value is 38.5″, and the maximum pitch and roll angles offset are 19.8″ and 15.5″, respectively.

In the verification experiments mentioned above, only three targets are selected for calculation. It takes about 40 s average to aim at and lock one target. If three cooperative targets are used and locked, the total time needed is about 2 min, but if more than three targets are used, it would take more time. In fact, if more preparing time is allowed, the system could aim at and lock more targets, and this approach could achieve better results.

Compared with other methods listed in the introduction, the vehicle experiments’ results proved that the proposed approach in this paper has many advantages. By using theodolite, it can acquire quite high orientation information (20″), but the operation of theodolite requires long duration (usually more than 20 min), and theodolite cannot provide position information either; however, the proposed approach in this paper is easy to operate and can obtain the same orientation accuracy as theodolite within 3 min as well as high accuracy positioning information.

The positioning accuracy of medium-accuracy of INS/DR is about several hundred meters while the orientation accuracy is about 3′. Compared to the INS/DR integration, the proposed approach improves the positioning and orientation accuracy significantly, with positioning error of less than 1 m and orientation error of about 20″. If the same positioning and orientation accuracy were to be acquired by INS/DR, the drifts of gyros employed in INS must be less than 0.001°/h, which will greatly increase the cost. However, the proposed approach only needs a rough position and orientation as an algorithm’s iterative initial value by INS/DR, so the cost can be reduced substantially.

The positioning accuracy of GNSS can be less than 1 m, but the GNSS signals are easy to interfere with and are requested not to be used during wartime. In addition, in order to achieve 20″ orientation information by GNSS, multiple GNSS antennas and long base line (several decameters) are needed; it is not easy to operate in practical situation. Therefore, the proposed approach has obvious advantages in positioning and orientation accuracy, rapidity, autonomy and cost; consequently, the approach has considerable potential in rapid and accurate positioning and orientation applications for land missile-launching vehicles.

## 5. Conclusions

The paper presented a new approach to get the land vehicle’s accurate position, azimuth and attitude rapidly. It uses a BODP to aim at and lock no less than three pre-set cooperative targets, whose accurate position coordinates are measured beforehand. The vehicle’s accurate position and orientation through the rough position and orientation provided by vehicle based INS/DR and at least three couples of azimuth and pitch angles measured by BODP while aiming at and locking cooperative targets are then calculated. It does not have high performance requirements for vehicle-based INS. Meanwhile, it does not depend on GNSS when determining vehicle’s position, attitudes and azimuth; thus, it is autonomous and difficult to interfere. The approach’s effectiveness is verified and evaluated by both simulation and vehicle experiments. The results indicate that it can achieve positioning and orientation accuracy of 0.2 m and 20″ respectively in 3 min; thus, it has high potential engineering values. Still, it has lots of work to do, including the influence of targets and vehicle distribution analysis, influence of vehicle based INS/DR error on the results analysis, *et al*. These are the next projects for our team to accomplish in the near future.
